# Biplane opening wedge high tibial osteotomy with a distal tuberosity osteotomy, radiological and clinical analysis with minimum follow-up of 2 years

**DOI:** 10.1186/s40634-019-0176-6

**Published:** 2019-02-28

**Authors:** Juan Erquicia, Pablo Eduardo Gelber, Simone Perelli, Federico Ibañez, Maximiliano Ibañez, Xavier Pelfort, Juan Carlos Monllau

**Affiliations:** 1 ICATME, Hospital Universitari Dexeus, UAB, Carrer de Sabino Arana, 5, Barcelona, 08028 Spain; 2 Department of Orthopaedic Surgery, Hospital de la Sta Creu i Sant Pau, UAB, Carrer de Sant Quintí, 89, Barcelona, 08026 Spain; 3 Department of Orthopaedic Surgery, Consorci Sanitari de l’Anoia, Hospital de Igualada, Av. Catalunya, 11, Barcelona, Spain; 4 Department of Orthopaedic Surgery, Hospital del Mar. Universitat Autònoma de Barcelona (UAB), Passeig Marítim, 25, Barcelona, 08003 Spain

**Keywords:** High tibial osteotomy, Open wedge tibial osteotomy, Biplanar tibial osteotomy, Tuberosity osteotomy, Patellar height, Tibial slope

## Abstract

**Background:**

High tibial osteotomy is an established and helpful treatment for unicompatimental osteoarthritis associated with varus deformity. However, asupratubercle high tibial osteotomy leads to a decrease in patellar height making the technique not suitable in case of concomitant patella baja. Moreover, this kind of osteotomy can change in situ forces at patellofemoral joint and the lateral patellar tilt. With the aim to widen the indication of high tibial osteotomy was proposed a biplane opening wedge high tibial osteotomy with a distal tuberosity osteotomy (B-OWHTO). This technique provide that the tibial tuberosity remains joined to the tibial metaphysis so as not to theoretically alter the patellar height. However, some Authors claim that BOWHTO could lead to an increase in tibial slope. The purpose of the present study was to assess the tibial slope, patella-femoral changes and axial correction as well as functional outcomes following a B-OWHTO.

**Methods:**

Patients operated on with a B-OWHTO and a minimum 24 months of follow-up were included. The mechanical alignment of the lower limb, patellar height, lateral patellar tilt and posterior tibial slope were calculated preoperatively, immediately after surgery and at the 24-month follow-up. The clinical results were evaluated using the Lysholm, Kujala and Hospital for Special Surgery knee scores. The possible postoperative development of patellofemoral pain or radiologic patellofemoral alteration was also evaluated.

**Results:**

Twenty-three patients were included with a mean follow-up of 33 months (range 27-41). The mechanical alignment of the lower limb shifted from a mean 9.3º ± 2.5 varus preoperatively to a mean 0.2º ± 2.2 valgus postoperatively. No changes in patellar height, lateral patellar tilt or in the posterior tibial slope were observed. The mean Lysholm and HSS scores improved from 68.3 ± 9.1 and 64.2 ± 5.2 preoperatively to 93.2 ± 2.1 and 94.1 ± 3.6 at final follow-up (*p* < 0.01). The mean Kujala score improved from 67.3 ± 9.8 to 86.4 ± 7.6 at final follow up (*p* < 0.01). No patients developed both radiological or clinical symptoms at patellofemoral joint.

**Conclusions:**

Open wedge high tibial osteotomy with a dihedral L-cut distal and posterior to the tibial tubercle accurately corrected axial malalignment without any change at patella-femoral joint or any modification to the posterior tibial slope while providing improved knee function at short-term follow-up. The radiographic as well as the clinical results support the use of this technique to treat medial compartment knee osteoarthritis and varus malalignment in young and middle-aged patients with a normal-to-low patellar height.

**Level of evidence:**

Case series with no comparison group, Level IV.

## Background

High tibial osteotomy (HTO) is an established treatment for patients with medial compartment knee osteoarthritis (OA) and varus malalignment (Poignard et al. 2010). Open wedge high tibial osteotomy (OWHTO) has gained in popularity over recent years as a viable alternative to the traditional lateral closed wedge osteotomy. This technique has several advantages over the lateral closed wedge osteotomy. They include avoiding a fibular osteotomy and the risk of peroneal nerve complications (Gaasbeek et al. 2010), smaller surgical exposure without muscle detachment, easier and more precise correction that can be change even after the osteotomy cut (Bito et al. 2009; Hankemeier et al. 2010). Furthermore, it can make an eventual future knee replacement easier not only due to the location of the skin approach but more because an OWHTO decreases the metaphyseal deformity caused by a closed wedge (Hui et al. 2011).

However, OWHTO has some potential disadvantages for the patellofemoral joint. Firstly, because a supra-tubercle osteotomy can lower the height of the patella, which is particularly true when a large correction is required (Amzallag et al. 2013). As a consequence, the use of an OWHTO is not usually recommended in cases of *patella infera. (Schallberger* et al. 2011*;* *Lobenhoffer* et al. 2009*)* However, it should be kept in mind that the origin of *patella infera* is likely multifactorial and so even patients with a preoperative normal-to-low patellar height could end up with this condition. Additionally, OWHTO can increase patellofemoral contact pressure and cause patellofemoral degeneration overtime even when patellar height has not been modified. (Stoffel et al. 2007) For those reasons, some surgeons advise against OWHTO when patellofemoral pain or patellofemoral chondral alterations are present even in patients with normal patellar height (Il et al. 2017; Kim et al. 2016; Kloos et al. 2018). A study recently has shown that an alteration of the axial alignment of the patella with a change of the lateral patellar tilt can be observed with a standard OWHTO. This fact could contribute to explaining the changes described before (Lee et al. 2016).

A second possible drawback of supra-tubercle OWHTO is its tendency to increase the sagittal tibial slope (Akizuki et al. 2008). Increasing the tibial slope may affect antero-posterior translation of the tibia as well as the in-situ forces on the anterior cruciate ligament.

With the aim of solving these potential problems and so broaden OWHTO indications, some authors still support the use of the standard infra-tubercle HTO in these cases (Shim et al. 2013). However, moving the tibial osteotomy distally reduces the total surface of cancellous bone at the osteotomy site and increases the risk of non-union. Several authors have already reported superior clinical outcomes of traditional supra-tubercle OWHTO over infra-tubercle HTO due to lower non-union rates (Noyes et al. 2006).

The further solution suggested by some authors in previous studies to deal with these issues is to perform a modification of the standard technique: a biplane OWHTO with a distal tuberosity osteotomy (OWHTO-B). This technique provide that the tibial tuberosity remains joined to the tibial metaphysis so as not to theoretically alter the patellar height without increasing the patellofemoral pressions (28) or even diminishing them (Kloos et al. 2018).

The aim of this study was then to assess the tibial slope, any patello-femoral change and axial correction as well as functional outcomes following a OWHTO-B. It was hypothesized that while providing good clinical results, this technique would accurately correct the axial malalignment without producing patello-femoral radiological alterations and keeping the posterior tibial slope unchanged.

## Methods

A retrospective analysis was performed of patients who had undergone an OWHTO-B for symptomatic medial osteoarthritis between 2012 and 2014 at the authors’ institution. Patients who did not have the corresponding radiographies before surgery, immediately after surgery and 24 months after the index procedure were also excluded. All the radiographic studies had to be performed at the index institution.

Like for every tibial osteotomy, the general indications for surgery were the presence of a varus tibial deformity associated with symptomatic narrowing of the medial compartment space, the absence of a flexion contracture, a flexion range of motion of at least 90° and the failure of other conservative treatment. OWHTO-B was particularly indicated when possibility of postoperative Caton Deschamp Index (CDI) less than 0.8 was present due to the risk of excessive and somehow unpredictable degree of patellar lowering: 1) in cases of a CDI less than 0.8, 2) in cases of CDI between 0.8 and 1, but a planned correction greater than 10° and, as extended indication, 3) in case of combined varus malalignment and patellofemoral pain or radiological patellofemoral alterations with a normal CDI. This is due to its ability to decrease the patellofemoral joint pressures (Kloos et al. 2018).

Contraindications were severe bone loss in the femoral condyle or tibial plateau, the presence of rheumatoid arthritis or infectious arthritis, the presence of prominent osteoarthritis in the lateral compartment and markedly limited joint motion.

The clinical research ethics committee of our institution approved the study (protocol number HTOL 2017–01). All the patients signed informed consent to participate in the study as well as for the evaluation and publication of their results.

### Preoperative study

**Fig. 1:**
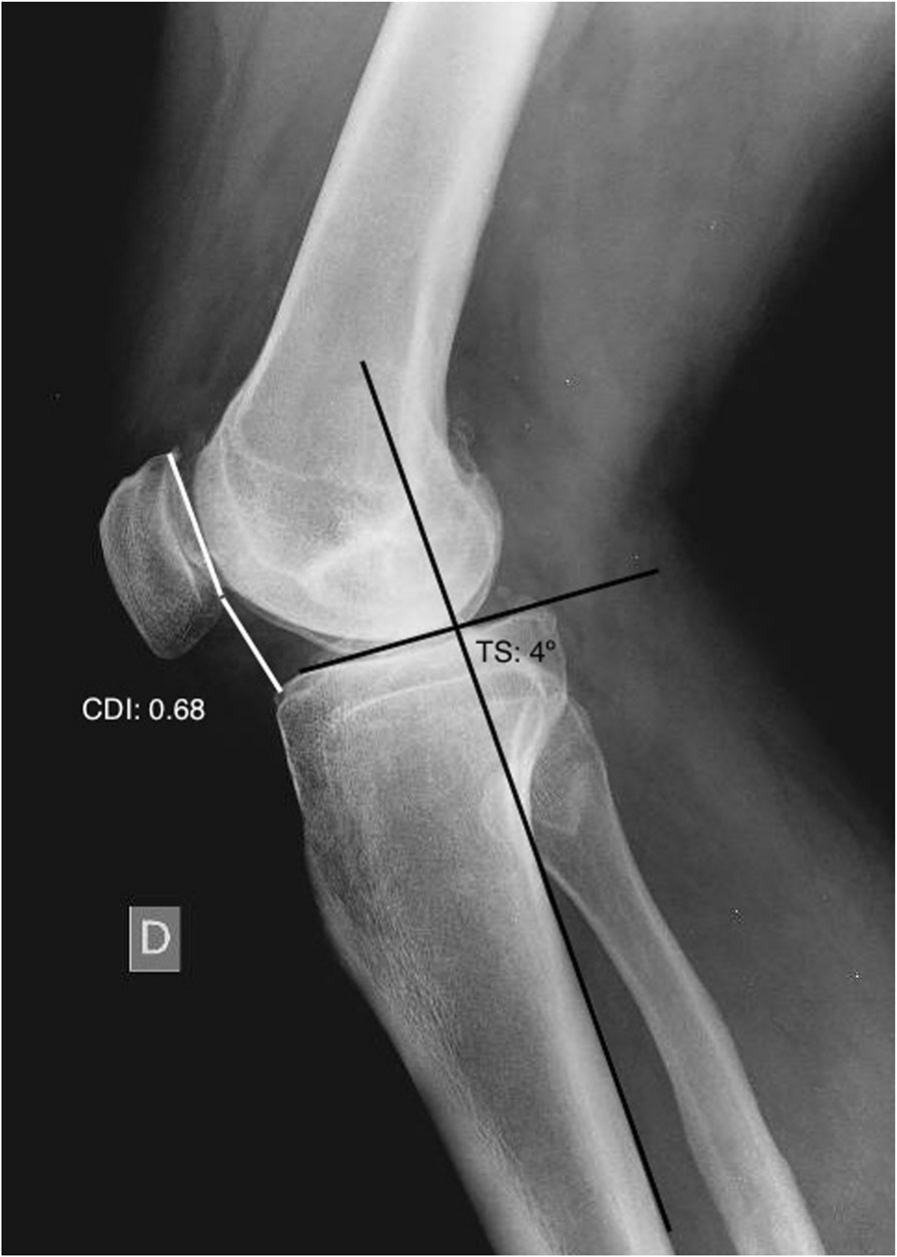
Measurement of the Caton-Deschamps index and the posterior tibial slope. *CDI:* Caton- Deschamps index, *TS:* tibial slope

**Fig. 2:**
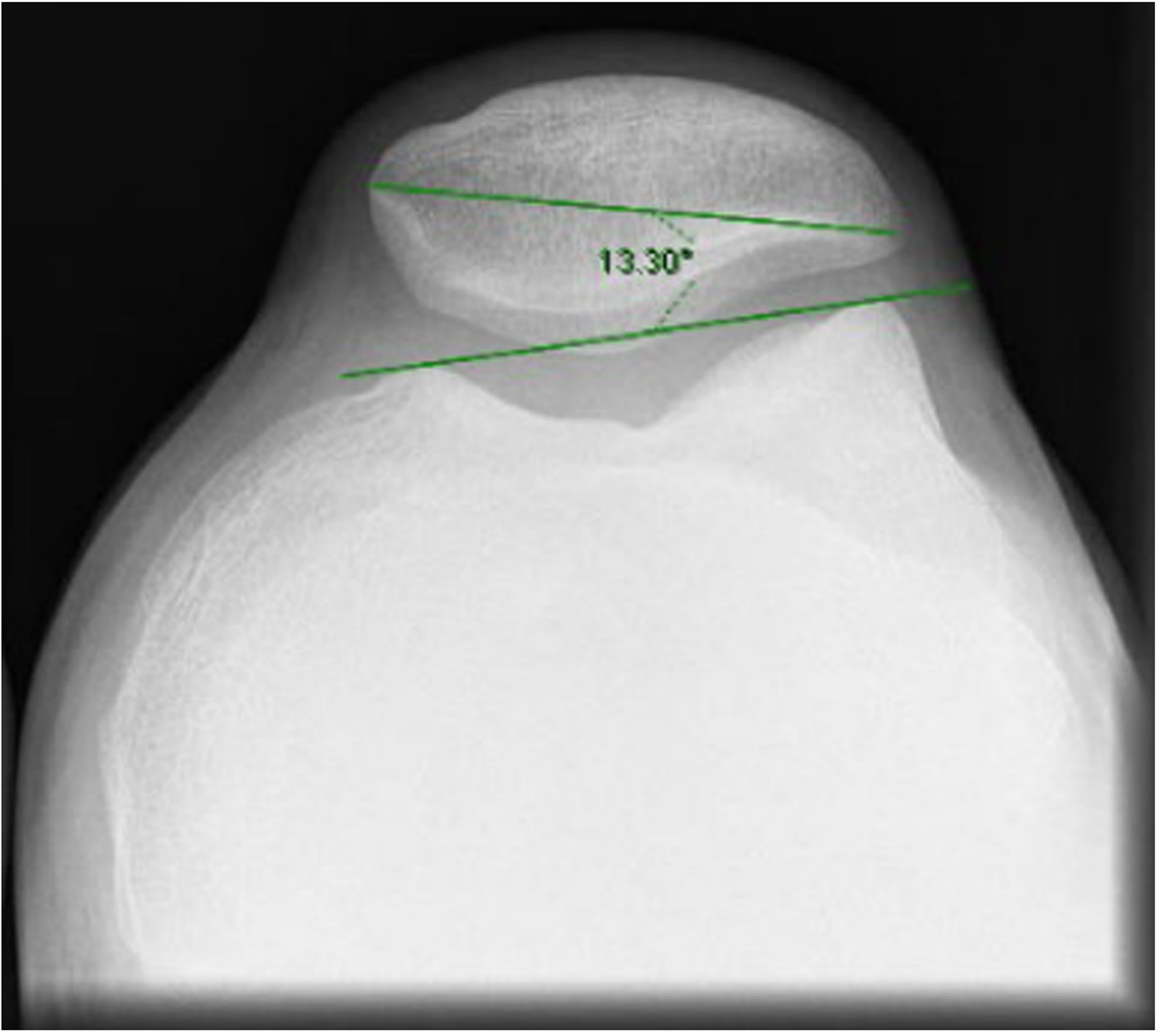
Measurement of the Lateral Patellar Tilt

- Full-weight-bearing long-leg standing anteroposterior radiographs were performed to determine the angle of the extremity and the desired degree of correction. A postoperative anatomic femoral-tibial axis of 5–8° valgus and a mechanical axis around the Fujisawa point was the goal (Habata et al. 2006)*.*
- The Schuss or Rosenberg radiographic view (Rosenberg et al. 1988) was used to evaluate the joint line space.
- A lateral non-weight-bearing radiography of the knee at 30° of flexion was used to measure the posterior tibial slope and the patellar height. The angle of the posterior tibial slope was determined based on a line passing through the posterior cortex of the tibia and another parallel to the joint slope (Brazier et al. 1996). For patellar height, the CDI was measured as previously described (Caton et al. 1982) (Fig. 1).
- A Merchant view (skyline view with knee flexed to 45°) was used to calculate the lateral patellar tilt (LPT) and the severity of patellofemoral arthritis. The latter was classified into 4 stages according to Merchant et al. (Kim and Joo 2012) (Fig. 2)

Two observers, blind from the aim of the study, performed all the radiographic measurements. They were carried out using the PACS system (Centricity Enterprise Web V3.0; GE Healthcare).

### Surgical technique

Two senior surgeons performed all the procedures. Once an arthroscopic evaluation of the knee had been done, a 6–7 cm-longitudinal approach mid-way between the tibial tuberosity (TT) and the posteromedial border of the tibia was performed. The semitendinosus and gracilis tendons were released and the superficial medial collateral ligament was freed from its distal insertion. The patellar tendon was identified and protected with a retractor.

#### Osteotomy

**Fig. 3:**
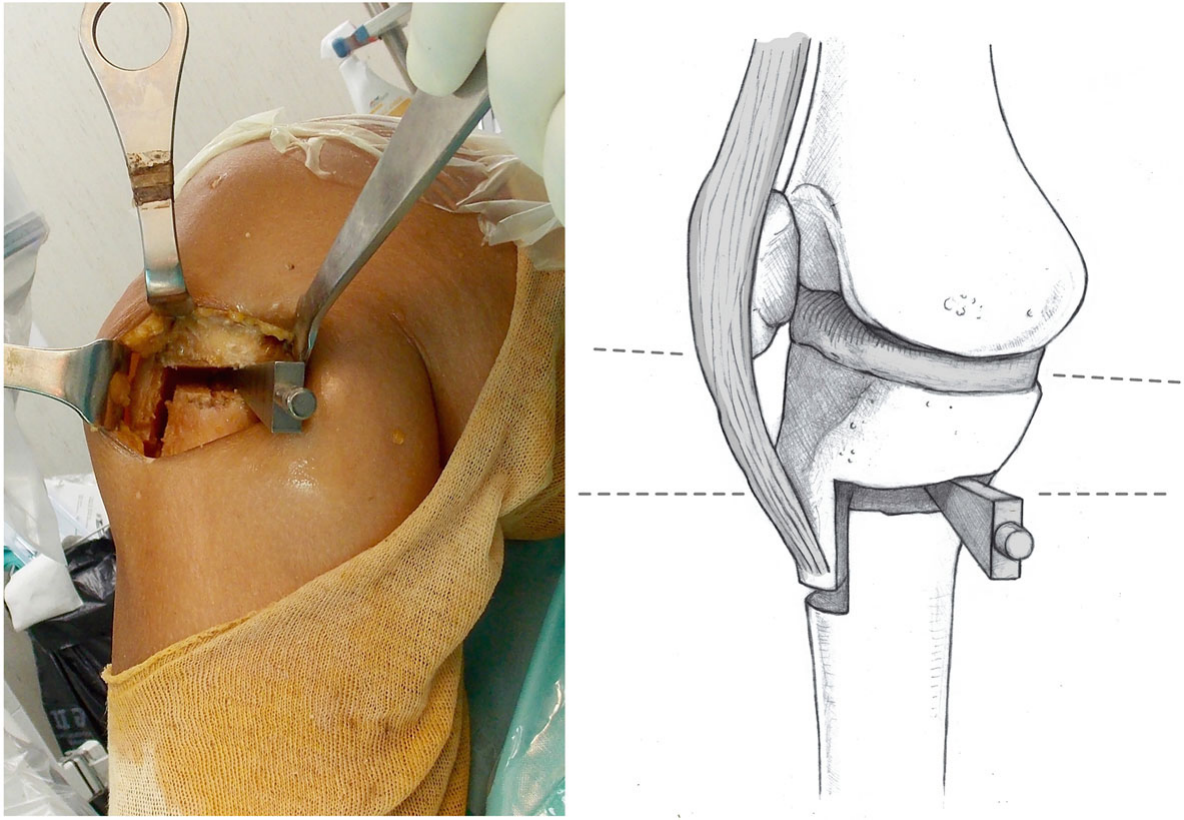
Graduated wedge at the level of the posterior tibial cortex

**Fig. 4:**
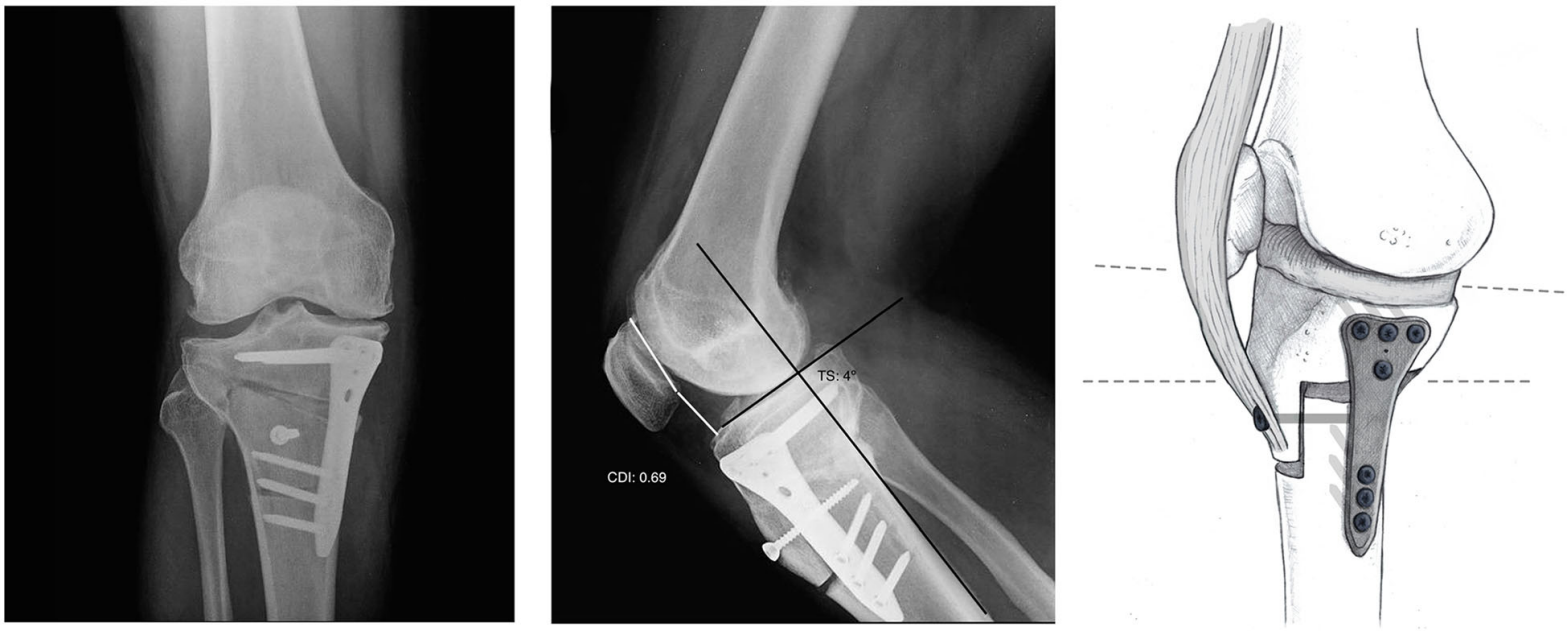
Postoperative aspect, without significant modifications in patellar height or the posterior tibial slope

The OWHTO-B has two different osteotomy planes. While the horizontal cut is similar to the standard OWHTO, the vertical part of the osteotomy is performed posterior to the tibial tubercle. Under fluoroscopic control, one 2.4-mm guiding Kirschner wire was placed in the medial cortex of the tibia at the metaphyseal-diaphyseal transition zone with the aim of tipping the fibular head in a proximal and posterolateral direction. The K-wire guided the sagittal cut performed in the posterior two thirds of the tibia. It is crucial to maintain perfect perpendicularity to the main axis of the bone on the sagittal plane to avoid tibial slope modifications. This section of the osteotomy ended 1 to 2 cm medial to the lateral border of the tibia. The second section of the osteotomy was vertical in the coronal plane and extended 3 to 4 cm distally. Thus, the TT together with the proximal segment of the osteotomized tibia was maintained. A thickness of 10 mm of the TT should be maintained in the most proximal part to minimize the risk of fracture. The desired correction is achieved using a metallic wedge introduced in the posterior-most part of the osteotomy site, thereby creating a trapezoidal gap to avoid an increase in the tibial slope (Fig. 3). To prevent anterior tilting of the TT leading to an increase in the posterior tibial slope, one or two anteroposterior cortical screws fixing the TT were secured before the osteotomy plate was placed. Then, the locking LOQTEQ®HTO plate (Berlin, Germany), was accordingly fixed (Fig. 4). Finally, the osteotomy gap was filled with an iliac crest allograft*.*

### Rehabilitation protocol

Patients started continuous passive motion of the knee as well as isometric quadriceps strengthening exercises immediately after surgery. During the first 3 weeks, patients were only allowed toe-touch partial weight-bearing. Then, progressive weight bearing as tolerated started. Full weight bearing was allowed after week 6.

Complications were recorded during the study period, which had a minimum follow-up of 24 months.

The same preoperative radiological studies were carried out at the latest follow up.

### Clinical assessment

Clinical and functional follow-up included the Lysholm, Kujala and Hospital for Special Surgery (HSS) knee scores. The physical examination as well as the functional evaluation of every patient was performed preoperatively and at final follow-up by a single sports medicine surgeon who was independent of the study. Attention was paid to the clinical evaluation of any postoperative patellofemoral pain manifested by patients.

### Radiological follow up

In every case a standard X-ray protocol was obtained preoperative and at 1–6–12-24 months postoperative. The protocol comprised a full-weight-bearing long-leg standing anteroposterior radiographs, a lateral non-weight-bearing radiography of the knee at 30° of flexion, a Merchant view. Femoral-tibial angle, posterior tibial slope, CDI, LPT and degree of patellofemoral arthritic degeneration were collected. Immediately postoperative was not obviously possible to obtain a full-weight-bearing long-leg standing X-ray as well as a Merchant view. Only standard anteroposterior and lateral non-weight-bearing radiography of the knee at 30° of flexion were obtained.

CDI and posterior tibial slope were collected starting from the immediate postoperative radiographs.

LPT, femoral-tibial angle and severity of patella-femoral arthritis were collected starting from the 1-month follow-up radiographs.

### Statistical analysis

Continuous variables are presented as means, standard deviations (SD), maximums and minimums. Categorical variables are presented as percentages and frequencies.

For analysis of the repeated measures, Bonferroni’s correction was used to identify differences between baseline and follow-up radiographic measurements.

Because of the small sample number, statistical tests were not utilized to evaluate normality. Instead, the assessment was performed with non-parametric equivalents, which showed no discrepancies in terms of significance. The inference in continuous variables was calculated with the paired-samples T-test and their results are presented with their 95% confidence interval (95% CI). Interobserver variability was analyzed with the variance test. The level of significance was set at 5% (α = 0.05), a bilateral approximation. All the analyses were performed with the SPSS 19 (SPSS Inc., Chicago, Illinois).

## Results

During the studied period 33 L-OWHTO were performed. Ten patients were excluded because some of the necessary postoperative images were lacking. Twenty-three patients (15 males and 8 females) with a mean age of 42.7 years (range 20.1 to 54.3 years) were finally included. The median body mass index was 27.4 (range of 21.6–32.7). The mean follow up was 33 months (range 27–41) Concomitant surgical procedures were: 1 anterior cruciate ligament reconstruction (4.3%), 8 meniscectomy (34.7%), 4 microfractures at medial condyle (17.4%).

### Radiological assessment

**Fig. 5:**
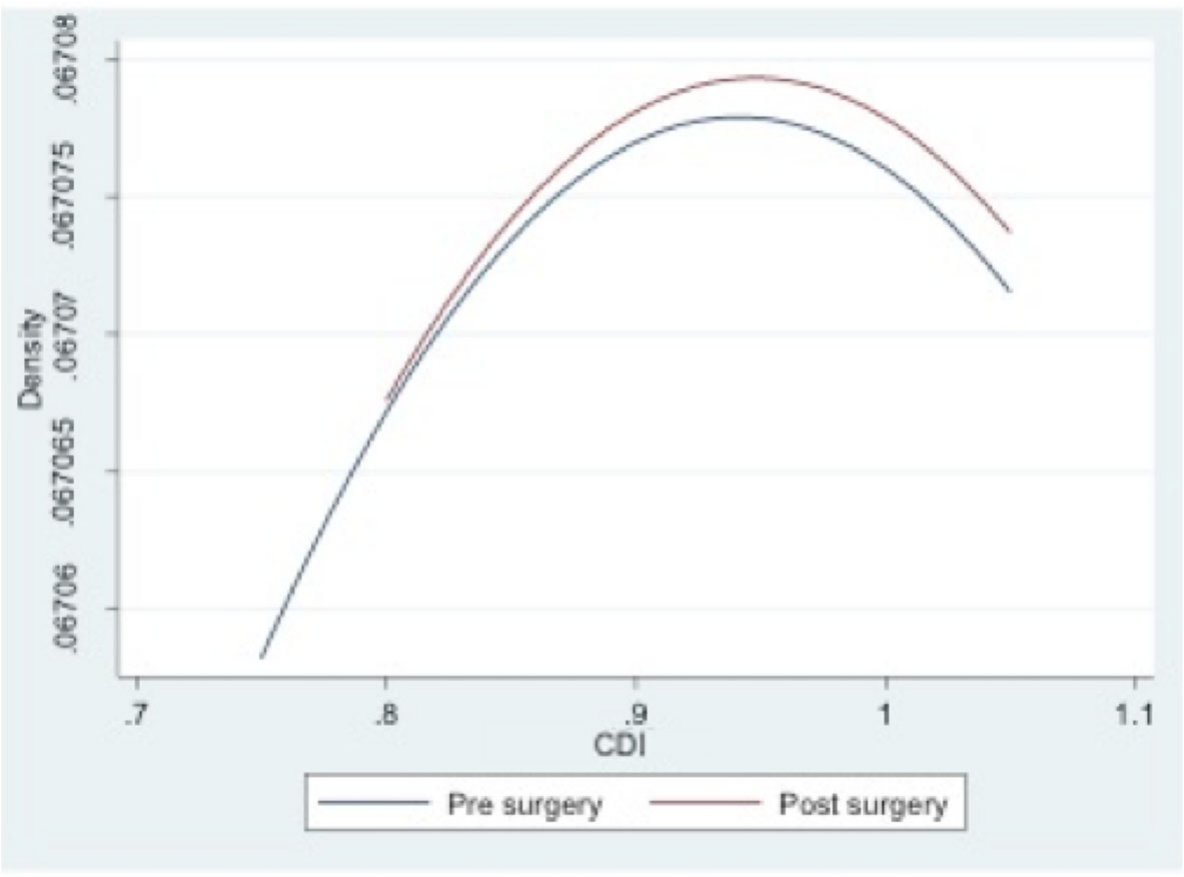
Pre- and postoperative Caton-Deschamps indexes

**Fig. 6:**
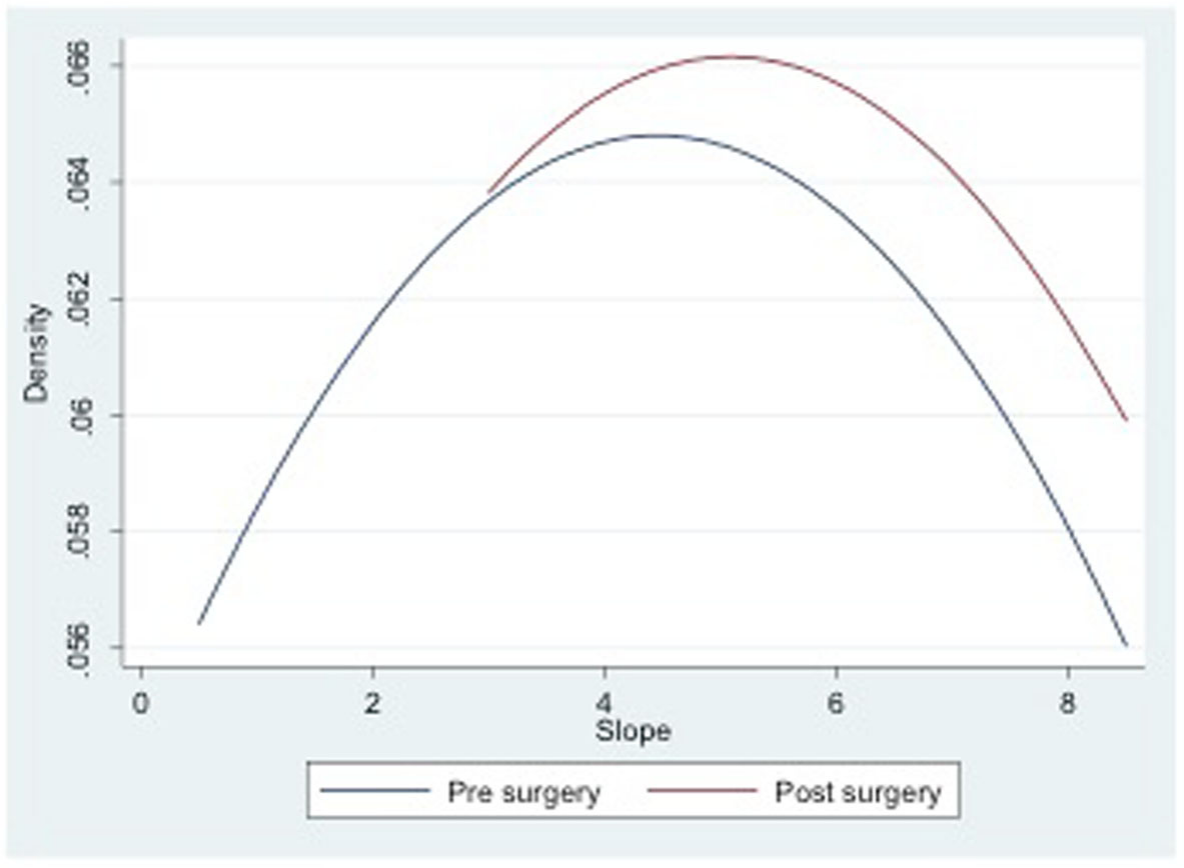
Pre- and postoperative tibial slopes

The mechanical alignment of the lower limb shifted from a mean varus of 9.3° ± 2.5 preoperatively to a mean valgus of 0.2° ± 2.2 four weeks after surgery and to a mean valgus of 0.4° ± 2.6 at the 24-month follow-up assessment (Table 1). The osteotomy did not alter neither the posterior tibial slope nor the patellar height in the immediate postoperative radiography (Table 1 and Figs. 5 and 6). No differences were noted neither in lateral patellar tilt at 1-month postoperative X-ray (Table 1). All these parameters remained unchanged at the 24-month follow-up (Table 1). Radiographically, most of the patellofemoral joints (20 out of 23) were graded I or II in the Merchant stage system. Merchant grades of the patellofemoral joints were not significantly different at preoperative evaluation and at latest follow up. There were no differences in the measures calculated for the two observers.

**Table 1:** Statistical analyses between preoperative and final follow-up results

	Preoperative mean score ± SD	Postoperative mean score ± SD	24 months postoperative mean score ± SD	Comparison of preop and postop (*P* value)	Comparison of preop and 24 months postop (*P* value)	Comparison of postop and 24 months postop (*P* value)
Mechanical axis	9.2° ± 2.5°	−0.2° ± 2.2°	−0.4° ± 2.6°	< 0.001	< 0.001	NS
Patellar height	0.94 ± 0.1	0.95 ± 0.08	0.95 ± 0.12	NS	NS	NS
Tibial slope	4.4° ± 2.1°	5.1° ± 1.4°	5.2° ± 1.4°	NS	NS	NS
Lateral Patellar Tilt	13.1° ± 2.9°	12.9° ± 3.2°	12.8° ± 3.7°	NS	NS	NS

*SD* standard deviation, *NS* not significant

Postoperative score is intended as immediate postoperative for patellar height and posterior tibial slope and 1-month follow-up for tibial slope ant lateral patellar tilt

### Clinical results

The mean Lysholm and HSS scores improved from 68.3 ± 9.1 and 64.2 ± 5.2 preoperatively to 93.2 ± 2.1 and 94.1 ± 3.6 at final follow-up (*p* < 0.01). The mean Kujala score improved from 67.3 ± 9.8 to 86.4 ± 7.6 at final follow up (p < 0.01). Preoperatively 5 out of 23 patients reported mild patellofemoral pain, at the final follow up no patients complained about anterior knee pain.

Only 2 out of the 23 patients included in the study showed complications. One patient had a pulmonary thromboembolism. This patient had had similar episode two decades before. It was successfully treated with oral anticoagulants. Another patient required hardware removal 28 months after surgery. No problems relative to unions or infections were observed.

## Discussion

The most important finding of the present study was that a OWHTO-B accurately corrected varus axial malalignment in patients with mid-to-low patellar height without causing any patellofemoral change or increasing the posterior tibial slope. The patients also had improved outcomes in the evaluated functional outcomes. All these findings confirmed our hypothesis. The rate of complication was low and all the osteotomies healed without delayed union.

Traditional OWHTO techniques cause distalization and lateralization of the TT, resulting in a decrease of patellar height as suggested by Goutallier using a mathematical model (Goutallier et al. 1979)*.* In a recent study, Amzallag et al. showed that patellar height decreased more than 20% in one-third of patients after an OWHTO. (Amzallag et al. 2013)

These changes would negatively affect the functional results of the osteotomy. Likewise, several recent studies confirmed the advantages of the closed-wedge HTO over the OWHTO in relation to patellar height preservation (Bin et al. 2016; Gaasbeek et al. 2004; El-Amrani et al. 2010; Ozel et al. 2015; Brinkman et al. 2008; Noyes et al. 2005; Stoffel et al. 2007; Ferner et al. 2018). This is even more important in higher axial deviations as a correlation between the degree of axial correction and the lowering of the patella has been shown (Amzallag et al. 2013). With the OWHTO-B technique, the tibial tuberosity remains attached to the proximal fragment of the osteotomy. Subsequently, the patellar height remained unchanged. The OWHTO-B is not intended to increase patellar height as it does not move the patella upwards. Therefore, this technique is not recommended in cases of a Caton Deschamp Index less than 0.6. Instead, a combined HTO and a separate osteotomy of the TT to heighten the tuberosity is indicated to handle the varus deformity and the patellar height.

Other authors have already shown some similar modifications to the traditional OWHTO technique in order to avoid modifications of patellar height. Gaasbeek et al. (Gaasbeek et al. 2004), in a study with 17 patients, showed the results of an OWHTO preserving the TT attached to the proximal tibia and also fixing the TT with an anteroposterior screw. However, the authors did so after the osteotomy plate was fixed. Then, this anteroposterior screw could not have prevented any eventual anterior tilting of the proximal fragment. In addition, the authors did not measure the tibial slope changes. They also had two complications. One of the patients had a fracture of the TT and another patient had a delayed union. Conversely, no complications related to the technique were observed in the current study. It is likely that the difference in the sequencing of the osteotomy’s fixation provided better contact between the tibial tuberosity and the distal tibial fragment.

To avoid modifications in patellar height and the tibial slope, Shim et al. (Shim et al. 2013) analyzed the results of an OWHTO distal to the TT. Despite achieving acceptable clinical and radiological results and because of the possibility of delayed union, they suggested the use of this technique only for those patients with an open physis and/or require minor corrections, have a body mass index below 25 and that the osteotomy gap be filled with autogenous iliac crest bone graft.

Standard OWHTO is contraindicated in patients with patella *infera*. The Caton-Deschamps index defines patella *baja* as those between 0.6 and 0.8. Those below 0.6 are considered a patella *infera*. However, there is no consensus on its threshold in terms of deciding whether to perform the OWHTO as the procedure itself decreases patellar height. In a retrospective level IV evidence study of patients operated on with an OWHTO, El Amrani et al. (El-Amrani et al. 2010) observed that the worse functional results were associated with postoperative patella *infera*. Gaasbeek et al. (Gaasbeek et al. 2004) also found a significant reduction in the CDI after OWHTO. All the patients showed lowering of the patella after the osteotomy and a direct correlation was found between the degree of wedge opening and the decrease in patellar height. In the present study, the OWHTO-B was performed when possibility of postoperative CDI less than 0.8 was present due to the risk of excessive and somehow unpredictable degree of patellar lowering.

Kim et al., using an SPET/TC evaluation, have shown that an increased signal activity around the patellofemoral joint was present after an OWHTO even when a low decrease of postoperative Blackburne-Peel ratio was found (Kim et al. 2016). Kim et al. reported that 21.9% of the patients developed patellar OA and 41.2% developed a trochlear OA after OWHTO. Their findings were based on a second look arthroscopy made at 21 to 32 months of follow-up (Il et al. 2017). The prominent finding was that no correlation of this evolution with patellar height was found. The conclusion could be that even when the patellar height has not been changed, alterations in patellofemoral pressure forces happen after a OWHTO. That is also supported by a recent cadaveric biomechanical study (Kloos et al. 2018). In any case we must take into account that always a large deformity correction in medial open-wedge high tibial osteotomy may cause a degeneration of patellofemoral cartilage. (Otakara et al. 2019) in the present study an arthroscopic second look wasn’t provide, but Kim in his study showed as well that 11.4% of the cases had postoperative anterior knee pain and among them all showed progressed OA on second look arthroscopy. Postoperative anterior knee pain was related to the ICRS grade of the patellofemoral joint at the time of second-look arthroscopy. In our study no patients complained about patellofemoral pain at the last follow up, neither the patients who reported patello-femoral discomfort before surgery. Finally, in their OWHTO series, Lee et al. have shown postoperative statistically significant changes in lateral patellar tilt (Lee et al. 2016). Even if they reported that this data has no clinical impact at 2 years follow-up, this mechanical alteration could as well explain patellofemoral cartilage subclinical alteration and patellofemoral pain at a longer term follow up. In the present study no significant alterations in postoperative lateral patellar tilt was detected at minimum 24 months follow up.

Several studies have shown that HTO might result in tibial slope changes. (Lu et al. 2018; Wu et al. 2017) in general, the posterior tibial slope increases after open-wedge high tibial osteotomies and decreases after the closed-wedge procedures. The increase in the tibial slope might alter in situ cruciate ligament forces and influence knee stability (Ozel et al. 2015)*.* Changes to the tibial slope might also accelerate the process of joint degeneration due to alterations in knee kinematics and biomechanics as it leads to higher tibiofemoral contact pressure on the anterior portion of the tibial plateau and decreased pressure on the posterior femoral condyle (Brinkman et al. 2008)*.*

To maintain the posterior slope, it is suggested that the anterior opening of the osteotomy site should be half of the posterior opening of the wedge (Noyes et al. 2005). The current technique, on the other hand, avoids any increment in the tibial slope by creating a trapezoidal gap and first fixing the TT with an anteroposterior screw.

The main limitation of this study was the low number of patients and the lack of control group. However, it is the largest reported series on such technique. Another limitation is that the short follow-up did not allow for the assessment long-term functional and radiological outcomes. Notwithstanding, the purpose of the investigation was to rule out any immediate radiological flaws of this relatively new technique.

For that reason, we excluded patients with an incomplete postoperative radiographic evaluation. However, the latter could be considered as a selection BIAS.

## Conclusions

Biplane OWHTO with a distal tuberosity osteotomy accurately corrected axial malalignment without changing patellar femoral height, lateral patellar tilt or the posterior tibial slope while providing improved knee function at short-term follow-up.

## Abbreviations

CDI: Caton Deschamp Index
HSS: Hospital for Special Surgery
HTO: High tibial osteotomy
LPT: Lateral patellar tilt
OA: Osteoarthritis
OWHTO: Open wedge high tibial osteotomy
OWHTO-B: Biplane opening wedge high tibial osteotomy with a distal tuberosity osteotomy
SD: Standard deviation
TT: Tibial tubercle
